# Supplementary material for: Knowledge, Attitudes, and Practices Related to Mold Remediation Following Hurricane Ida in Southeast Louisiana
Source: Int J Environ Res Public Health. 2024 Oct 25;21(11):1412. doi: 10.3390/ijerph21111412 (PMC11594125; doi:10.3390/ijerph21111412)
Supplement: Supplementary file 1 [file ijerph-21-01412-s001.zip › ijerph-3070063-supplementary.pdf]

Supplementary material:

Table S1. Differences between the post-Ida and post-Katrina outcome items and description of harmonization approach.

	Question wording				Coding for comparison analysis	
Variable	Post-Ida	Post-Katrina	Differences across the two surveys	Post-Ida	Post-Katrina
Knowledge	Which mask should you use when cleaning up mold?	Which mask should you use when cleaning up mold?	• Respirators meeting international standards (international respirators) (e.g., KF-94, KN-95), were available on the market after Hurricane Ida, but not after Hurricanes Katrina and Rita. These models were included among the response options in the post-Ida survey.<br>• Post-Hurricane Katrina respondents selected a single respirator or source control productwhile post-Ida respondents could select multiple.<br>• Post-Katrina responses were collapsed into four categories: none, dust or surgical mask, disposable or reusable respirator, or don't know. Information on the product models offered in the post-Katrina survey is not available, whereas we have detailed information on which product models respondents were offered in the post-Ida sample.	Only selecting a NIOSH Approved respirator was considered to align with recommendations (received a designation of 1). Selecting both a NIOSH Approved respirator and an international respirator or source control product, selecting an international respirator or source control product, or responding "none" or "don't know" were defined as a selection that does not align with recommendations (received a designation of 0).	Selecting the "disposable or reusable respirator" category was considered to align with recommendations (received a designation of 1). Selecting any of the remaining three categories (none, dust or surgical mask, or don't know) was defined as not aligning with recommendations (received a designation of 0).
Attitudes	Do you think you are personally at risk of getting sick from mold?	Do you think you are personally at risk of getting sick from mold?	None.	Yes (1), No (0), Don't know (0)	Yes (1), No (0), Don't know (0)

Practices	When removing mold have you used: Mask? (never, occasionally, often, always)<br>If occasionally, often, or always: Which mask do you use to clean up mold?	When removing mold have you used: Mask? (never, occasionally, often, always)<br>If occasionally, often, or always: Specify type.	• International respirators (e.g. KF-94, KN-95), were available on the market after Hurricane Ida, but not after Hurricanes Katrina and Rita. These models were included among the response options in the post-Ida survey.<br>• Post-Hurricane Katrina respondents selected a single respirator or source control productwhile post-Ida respondents could select multiple.<br>• In the post-Katrina sample, responses to the follow-up question (“specify type”) were collapsed into “mask” or “respirator” categories. Information on the specific product models offered in the post-Katrina survey is not available, whereas we have detailed information on product models respondents were offered in the post-Ida sample.	Only selecting a NIOSH Approved respirator was considered to align with recommendations (received a designation of 1). Selecting both a NIOSH Approved respirator and an international respirator or source control product, selecting an international respirator or source control product, or responding “none” or “don’t know” were defined as a selection that does not align with recommendations (received a designation of 0).	Selecting the “disposable or reusable respirator” category was considered to align with recommendations (received a designation of 1). Selecting any of the remaining three categories (none, dust or surgical mask, or don’t know) was defined as not aligning with recommendations (received a designation of 0).

Table S2. Respirators or source control products selected in response to the question, “Which mask(s) should you use to clean up mold?” among residents and workers in Southeast Louisiana.

Question	Overall N = 306	Resident N = 238	Worker N = 68
**Which mask(s) should you use to clean up mold?**			
Full Facepiece Respirator	162 (53)	126 (53)	36 (53)
Half Mask Respirator	170 (56)	123 (52)	47 (69)
Cup-Shaped N95 FFR	106 (35)	85 (36)	21 (31)
Cup-Shaped N95 FFR with Valve	131 (43)	100 (42)	31 (46)
Flat-Fold N95 FFR	34 (11)	21 (9)	13 (19)
KN95	28 (9)	23 (10)	5 (7)
KF94	10 (3)	6 (3)	4 (6)
Surgical Mask	11 (4)	11 (5)	0 (0)
Dust Mask	13 (4)	11 (5)	2 (3)
Cloth Mask	4 (1)	4 (2)	0 (0)
Gaiter	7 (2)	5 (2)	2 (3)
Bandana	5 (2)	3 (1)	2 (3)
No Mask	1 (<1)	1 (<1)	0 (0)
Don’t Know	9 (3)	8 (3)	1 (1)

Table S3. Respirator and source control product use for mold remediation among surveyed residents and workers in Southeast Louisiana who reported cleaning up mold (n = 194).

Question	Overall<br>n = 194	Resident<br>n = 126	Worker<br>n = 68
**Which mask(s) do you use to clean up mold?**			
NIOSH Approved Respirators			
Full Facepiece Respirator	23 (12)	12 (10)	11 (16)
Half Mask Respirator	59 (30)	29 (23)	30 (44)
Cup-Shaped N95 FFR	41 (21)	27 (21)	14 (21)
Cup-Shaped N95 FFR with Valve	40 (21)	21 (17)	19 (28)
Flat-Fold N95 FFR	7 (4)	2 (2)	5 (7)
Other Respirators			
KN95	21 (11)	16 (13)	5 (7)
KF94	3 (2)	1 (1)	2 (3)
Masks			
Surgical Mask	25 (13)	21 (17)	4 (6)
Dust Mask	10 (5)	7 (1)	3 (4)
Cloth Mask	6 (3)	4 (3)	2 (3)
Face coverings			
Gaiter	1 (1)	0 (0)	1 (1)
Bandana	4 (2)	3 (2)	1 (1)
None	26 (13)	25 (20)	1 (1)

Resident Questionnaire

1) **Prior to Hurricane Ida, had you ever experienced flooding of a home you lived in?**

Yes No Don't know

If #1 NO, skip to #3

2) **What was the nature or cause of your previous experience with flooding?**

Hurricane Delta	Hurricane Zeta	Hurricane Katrina	Other:	
Hurricane Barry	Hurricane Gustav	Plumbing issue		

3) **Prior to Hurricane Ida, had you ever experienced mold growth in your home?**

Yes No Don't know

If YES for #3:

4) **Were you able to fix the mold growth in your home that happened before Hurricane Ida?**

Yes No Don't know

5) **Was your home damaged by Hurricane Ida?**

Yes No

If NO for #5, SKIP to #9

6) **What was the extent of flooding to your home due to Hurricane Ida?**

None <3 feet 3-6 feet >6 feet Don't know

7) **What was the extent of mold growth in your home after Hurricane Ida?**

None <10 sqft 10-100 sqft >100 sqft Don't know

8) **How much time have you spent in a house with mold since Hurricane Ida?**

____________ Hours Days Weeks (circle as appropriate)

9) **Have you ever heard or seen any public messages from the state, like the health department, or government about cleaning up mold?**

Yes No Don't know

10) **What has been your main source of information about mold?**

Health dept	FEMA	Employer	Insurance Co.	Store
Internet	Facebook	Govt website	Google	Figured it out myself

Other:

11) **Which mask should you use when cleaning up mold? (show mask examples)**

1 2 3 4 5 6 7 8 9 10 11 12

None Don’t Know

12) **Which of these masks do you have at home, if any?**

1 2 3 4 5 6 7 8 9 10 11 12

None Don’t Know

13) **Which of these masks have you or would you consider using to protect yourself or others from COVID?**

1 2 3 4 5 6 7 8 9 10 11 12

None Don’t Know

14) **Would you know where to get a special mask called the N95 mask?**

Yes No Don’t know

15) **Have you purchased or received an N95 mask in the last few months?**

Yes No Don’t know

16) **How much would you spend for a special mask to use during clean-up of mold?**

$0 <$1 $1-5 $6-20 >$20 Don’t know

17) **How much bleach should be added to 1 gallon of water for cleaning mold?**

1 capful 1 ounce 1 cup Straight bleach, no water Don’t know

18) **Do you think mold can make people sick?**

Yes No Don’t know

19) **Do you think you personally are at risk of getting sick from mold?**

Yes No Don’t know

20) **Why do you think you are/aren’t at risk?**

21) **What would be the best way to get messages about mold to the public?**

Social media TV Radio Newspaper Door-to-door

Mailings Classes Stores Text messages

Other:

22) **Are you interested in learning more about cleaning up mold?**

Yes No Don’t know

23) Do you think you could still have a mold problem after all the visible mold is gone?

Yes No Don't know

24) Have you personally attempted to remove mold from your home?

Yes No Don't know

25) When removing mold have you used:

Gloves?

Never Occasionally Often Always Don't Know

Mask?

Never Occasionally Often Always Don't know

Which mask do you use to clean up mold? (show display)	1	2	3	4	5	6	7	8	9	10	11	12
	Other:											

Goggles?

Never Occasionally Often Always Don't know

Other:

Never Occasionally Often Always Don't know

26) Why do you use/not use this protective equipment?

27) What have you used or would you use to clean up mold?

Soapy water Bleach Ammonia Bleach mixed with ammonia Household cleaner:

Other:

28) Have you hired or are you considering hiring someone to remove your mold?

Yes No Don't know

29) Which of these images have you seen before, if any?

#1 #2 #3 (circle all that apply)

30) What's your gender?

Female Male Refused Other:

31) Age

32) Primary language

33) Secondary language

34) Zip code of residence

35) How would you describe where you live?

One-unit building, detached from any other building	One-unit building, attached to one or more buildings (e.g., townhome, duplex)	Building with two or more apartments	Manufactured/mobile home

36) Is your home:

Owned Rented Other:

37) Hispanic (circle all that apply)

No, not of Hispanic, Latino/a, or Spanish origin	Yes, Mexican, Mexican American, Chicano/a	Yes, Puerto Rican	Yes, Cuban	Yes, Another Hispanic, Latino/a, or Spanish origin

38) Race (circle all that apply)

White	Asian Indian	Korean	Native Hawaiian
Black or African American	Chinese	Vietnamese	Guamanian or Chamorro
American Indian or Native Alaskan	Filipino	Other Asian	Samoan
	Japanese		Other Pacific Islander

Other:

39) Occupation

40) Education

No high school	Some high school	High school graduate	Some college	College graduate	Graduate degree

41) Smoker

Current Former Never

42) Lung disease (e.g., asthma, COPD)

Yes No Don't know

Worker Questionnaire

1) **As part of your job, are you involved in mold clean-up activities? (On a volunteer basis or for wages)**

   Yes No

2) **Prior to Hurricane Ida, had you ever worked on flooded buildings before?**

   Yes No Don't know

3) **Prior to Hurricane Ida, had you ever worked on moldy buildings?**

   Yes No Don't know

4) **How much time have you spent in buildings with mold since Hurricane Ida?**

   # ____________ Hours Days Weeks (circle as appropriate)

5) **How long have you been working in mold cleanup?**

   # ____________ Years Months Days

6) **Have you ever heard or seen any public messages about cleaning up mold?**

   Yes No Don't know

7) **What has been your main source of information about mold?**

Health dept	FEMA	Employer	Insurance Co.	Store
Internet	Facebook	Govt website	Google	Figured it out myself

   Other:

8) **Which mask should you use when cleaning up mold? (show mask examples)**

   # ____________ None Don't know

9) **Which of these masks do you have at home, if any?**

   #s:

10) **Which of these masks have you or would you consider using to protect yourself or others from COVID?**

______________ None Don't know

11) **Would you know where to get a special mask called the N95 mask?**

Yes No Don't know

12) **Have you purchased or received an N95 mask in the last few months?**

Yes No Don't know

13) **How much would you spend for a special mask to use during clean-up of mold?**

$0 <$1 $1-5 $6-20 >$20 Don't know

14) **How much bleach should be added to 1 gallon of water for cleaning mold?**

1 capful 1 ounce 1 cup Straight bleach, no water Don't know

15) **Do you think mold can make people sick?**

Yes No Don't know

16) **Do you think you personally are at risk of getting sick from mold?**

Yes No Don't know

17) **Why do you think you are/aren't at risk?**

[ ]

18) **What would be the best way to get messages about mold to the public?**

Social media TV Radio Newspaper Door-to-door

Mailings Classes Stores Text messages

Other: [ ]

19) **Are you interested in learning more about cleaning up mold?**

Yes No Don't know

20) **Do you think you could still have a mold problem after all the visible mold is gone?**

Yes No Don't know

21) **When removing mold have you used:**

Gloves?

Never Occasionally Often Always Don't Know

Mask? Type: [ ]

Never Occasionally Often Always Don't know

Goggles?

Never Occasionally Often Always Don't know

Other:

Never Occasionally Often Always Don't know

22) Why do you use/not use this protective equipment?

23) What have you used or would you use to clean up mold?

Soapy water Bleach Ammonia Bleach mixed with ammonia Household cleaner:

Other:

24) What protective equipment has your employer provided for you?

Gloves Mask Goggles Don't know

Other:

25) Has your employer instructed you on how or when to use protective equipment?

Yes No Don't know

26) Have you had a test to see if your work mask fits to your face?

Yes No Don't know NA

27) Which of these images have you seen before, if any?

#1 #2 #3 (Circle all that apply)

28) What's your gender?

Male Female

29) Age

30) Primary language

31) Zip code(s) for work locations

32) Hispanic

	Yes, Mexican, Mexican American, Chicano	Yes, Puerto Rican	Yes, Cuban	No

33) Race

White	Black or African American	American Indian or Alaska Native	Asian Indian	Chinese	Filipino	Japanese
Korean	Vietnamese	Other Asian	Native Hawaiian	Guamanian or Chamorro	Samoan	Other Pacific Islander

34) Occupation

35) Education

No high school	Some high school	High school graduate	Some college	College graduate

36) Smoker

Current	Former	Never

37) Lung disease (e.g, asthma, COPD)

Yes	No	Don’t know
